# *Vibrio splendidus* Flagellin C-Induced Extracellular Trap Release Relies on AjTLR2 Recognition in *Apostichopus japonicus*

**DOI:** 10.3390/biom16081097

**Published:** 2026-07-27

**Authors:** Jiaqian Zhu, Yuxin Li, Yuxuan Liang, Jie Yu, Kaiyu Chen, Chenghua Li

**Affiliations:** 1 State Key Laboratory of Agricultural Products Safety, Ningbo University, Ningbo 315211, China; 2 School of Marine Science and Engineering, Qingdao Agricultural University, Qingdao 266109, China; 3 Laboratory for Marine Fisheries Science and Food Production Processes, Qingdao National Laboratory for Marine Science and Technology, Qingdao 266071, China

**Keywords:** Toll-like receptor, extracellular traps (ETs), innate immunity, echinoderms

## Abstract

Extracellular traps (ETs) are a novel host defense mechanism used to immobilize and eliminate invading microorganisms, and their formation depends on the recognition of foreign pathogens by membrane receptors. Previous studies have demonstrated that *Vibrio splendidus* flagellin can induce the release of ETs in coelomocytes of the sea cucumber *Apostichopus japonicus*, yet the underlying regulatory mechanism remains unclear. Here, we identify another Toll-like receptor (TLR) homolog, AjTLR2, in *Apostichopus japonicus*, which is composed of an extracellular LRR domain, a transmembrane domain, and an intracellular TIR domain. As a membrane receptor, AjTLR2 is upregulated upon infection with *Vibrio splendidus* AJ01, which is isolated from diseased *Apostichopus japonicus*. The extracellular LRR domain exhibits binding activity toward LPS, PGN, and MAN. In addition to these ligands, AjTLR2 recognizes flagellin C of AJ01 (AJ01-FliC), whereas other AjTLRs, such as AjToll and AjTLR3, do not. Further functional analysis reveals that knockdown of AjTLR2 results in a reduction in the typical weblike DNA structures of ETs, accompanied by a significant decrease in the expression of the ET-associated antimicrobial proteins H2A, H2B, and lysozyme. Furthermore, AjTLR2 knockdown similarly inhibits ET formation induced by recombinant AJ01-FliC protein. Mechanistically, the *Apostichopus japonicus* proto-oncogene tyrosine-protein kinase Src homolog (AjSRC), previously identified in our laboratory, is a downstream signaling molecule of AjTLR2 and is recruited via the TIR domain of AjTLR2. Knockdown of AjSRC also suppresses AJ01-FliC-induced ET formation. Collectively, our results indicate that the recruitment of AjSRC by AjTLR2 represents a potential regulatory pathway for AJ01-FliC induced ET generation.

## 1. Introduction

Throughout the evolutionary trajectory of animals, both vertebrate and invertebrate lineages have been subjected to persistent selective pressures from pathogenic microbes, a challenge that has driven the early emergence and conservation of the innate immune system [1]. Innate immunity responds rapidly to the invasion of pathogens and is therefore considered the first line of defense against pathogenic infections. The initiation of innate immune signaling generally relies on host-expressed pattern recognition receptors (PRRs) to recognize conserved microbial molecular structures termed pathogen-associated molecular patterns (PAMPs). Based on differences in the subcellular localization of PRRs, these receptors can be classified into three major categories. Secreted extracellular pattern recognition molecules include pentraxins, collectins and ficolins. Membrane-bound receptors located on the cell surface, include Toll-like receptors (TLRs, including TLR1, TLR2, TLR4, TLR5, TLR6 and TLR10), scavenger receptors, and C-type lectin receptors (CLRs), which primarily recognize extracellular pathogens and their cell wall components. Cytoplasmic receptors, include AIM2-like receptors (ALRs), NOD-like receptors (NLRs), and RIG-I-like receptors (RLRs), which mainly detect pathogen-derived components and damage-associated signals within the cytoplasm [2]. The TLR pattern recognition receptor family is an important class of PRRs and is currently considered one of the typical major receptors for pathogens in all metazoans.

So far, 13 TLRs have been characterized in mice and 11 in humans, comprising both transmembrane (TLR1/2/4/5/6/10) and cytosolic (TLR3/7/8/9) subfamilies [3]. Members of the TLR family share a conserved architectural framework, characterized by an extracellular segment rich in leucine-rich repeats (LRRs), a single transmembrane helix, and a cytoplasmic Toll/interleukin-1 receptor (TIR) domain [4]. The LRR domain located in the extracellular domain mainly plays a role in immune recognition, activating TLRs by rapidly recognizing PAMPs and DAMPs [4]. Although the extracellular segments of TLRs generally contain LRR domains, these TLRs have their own specific ligands. For instance, TLR2 functions as a versatile PRR by assembling into heterodimers with TLR1, TLR6, or non-TLR molecules such as CD36 [5]. Through these partnerships, it recognizes an extensive array of PAMPs, ranging from peptidoglycans (PGN) and bacterial lipoproteins to mycoplasma lipopeptides and fungal zymosan. In particular, TLR1/2 preferentially binds triacylated lipopeptides, whereas TLR2/6 selectively recognizes diacylated lipopeptides. TLR4 mainly recognizes bacterial lipopolysaccharides (LPS); TLR5 can specifically recognize flagellar proteins [3]. After identifying a PAMP, adaptor proteins MyD88 or TRIF are recruited through the TIR domain located in the intracellular segment [6]. Functioning as a central conduit for inflammatory signaling, MyD88 is recruited by all TLRs except TLR3 to propagate immune responses. MyD88 recruitment initiates a signaling cascade involving mitogen-activated protein kinases (MAPKs), including ERK, JNK, and p38, and the transcription factor NF-κB, which together induce the increased expression of IL-1β, IL-6, and TNF-α [7]. Activation of TLR3 and TLR4 facilitates TRIF recruitment, thereby triggering the TRIF-dependent pathway, driving the eventual phosphorylation and activation of key immune regulators, including NF-κB, MAPKs, and IRF3 [8]. In summary, the distinct array of adaptors recruited by individual TLRs serves to trigger varied transcription factors, facilitating context-appropriate and robust host defense mechanisms.

In addition to the classic TLR regulatory pathways mentioned above, TLRs are well documented to exert critical functions in mediating neutrophil extracellular traps (NETs), with reports suggesting that TLRs are necessary for vital NETs [9]. Due to their strong binding capacity for bacterial cell wall constituents and lipoproteins, TLRs are pivotal in mediating innate immunity initiated by NETs. Ting Wan et al. [10] found that TLR2 and TLR4 antagonists can significantly inhibit *S. aureus*-induced NETosis, while TLR2 and TLR4 agonists can enhance NETosis. Platelet TLR4 can induce binding between platelets and adherent neutrophils, leading to strong activation of neutrophils and formation of NETs [11]. Numerous studies have demonstrated the regulatory relationship between TLRs and NET formation in mammalian species, however the regulatory pathways of ETs in invertebrates are currently unclear. Accumulating evidence has shown that *Vibrio splendidus* flagellin can induce the release of ETs in coelomocytes of *Apostichopus japonicus*, yet its specific regulatory mechanism has not been fully clarified [12]. Therefore, in this study, we identified AjTLR2 in *Apostichopus japonicus* as a receptor that recognizes *Vibrio splendidus* flagellin FliC via its extracellular LRR domain and recruits AjSRC through its intracellular TIR domain to regulate flagellin-induced ET production.

## 2. Materials and Methods

### 2.1. Animals

Healthy adult *Apostichopus japonicus* (average body weight: 125 ± 12 g) were obtained from Dalian Pacific Aquaculture Company. Prior to the experiment, all individuals were acclimatized in an oxygenated recirculating seawater aquaculture system, with a salinity of 28 and a water temperature maintained at 16 ± 1 °C. A mixed diet consisting of 8% fish meal, 22% seaweed powder, 50% starch, and 20% marine mud was supplied every two days. Residual and uneaten feed was promptly removed after each feeding, and water exchange was performed when necessary. Formal experiments were initiated after the sea cucumbers had fully acclimated to the laboratory conditions.

### 2.2. Recombinant Expression and Antiserum Preparation of AjTLR2

Amplification of the AjTLR2 extracellular domain was performed using gene-specific primers (AjTLR2-EX-F/R; Table S1). Subsequently, the recombinant extracellular domain of AjTLR2 (rAjTLR2-EX) was expressed in *E. coli* Rosetta cells and purified via a Ni-NTA Sepharose column (Roche), following established protocols [13,14].

Mouse polyclonal antibodies targeting AjTLR2 were generated according to established protocols described in prior literature [15]. Six-week-old female BALB/c mice were procured and employed for the purposes of this investigation. The purified recombinant protein served as the immunogen and emulsified with an equal volume of complete Freund’s adjuvant for the primary immunization or incomplete Freund’s adjuvant for booster immunization. Mice were immunized via multi-point subcutaneous injection on the back, with each mouse receiving 100 μg of antigen. Booster immunization was performed every 7 days after the primary injection, with a total of 3 immunization cycles. On the 7th day after the final immunization, blood samples were collected by orbital bleeding or cardiac puncture, and hemolysis was strictly avoided during operation. After 20 min of centrifugation (4000 *g*, 4 °C), the serum fraction was separated from whole blood. The resulting supernatant was divided into aliquots and frozen at −80 °C.

### 2.3. Phylogenetic Analysis

Phylogenetic trees were reconstructed using a maximum-likelihood (ML) approach. Protein sequences were aligned using MAFFT (version 7.526), trimmed with ClipKIT (version 2.12.2), and analyzed using IQ-TREE (version 2.4.0) with automatic model selection implemented in ModelFinder. Branch support was assessed using 1000 bootstrap replicates, and the resulting tree was rooted using the midpoint method. Internal nodes with bootstrap support values below 50% were collapsed into polytomies while retaining branch length information.

### 2.4. PAMP Binding Assay

The capacity to bind PAMPs was assessed using ELISA, following established protocols [14]. Three types of PAMPs were selected for the binding assay, including LPS derived from *Escherichia coli* 055:B5 (Sigma-Aldrich, St. Louis, MO, USA), PGN from *Staphylococcus aureus* (Sigma-Aldrich, St. Louis, MO, USA), and MAN from *Saccharomyces cerevisiae* (Sigma-Aldrich, St. Louis, MO, USA). All PAMPs were dissolved in 50 mmol/L carbonate-bicarbonate buffer (pH 9.6) to a final concentration of 0.2 mg/mL. A total of 30 μL of each PAMP solution was added to each well of a 96-well plate. The plate was gently tapped after loading to ensure uniform coverage of the well bottom, with five technical replicates set for each group, and incubated overnight at 4 °C. After discarding the PAMP solution, each well was rinsed twice with 200 μL aliquots of phosphate-buffered saline containing 0.05% Tween-20 (PBST, pH 7.2). Subsequently, 200 μL of 5% BSA solution (prepared with PBS) was added for blocking, and the sample plate was maintained at 37 °C for 1 h. Upon blocking, the BSA solution was discarded, and the wells were washed twice with 200 μL PBST. Next, 100 μL of rAjTLR2-EX protein at gradient doses (0, 3, 6, 9, 12, 15 μg) was added to each well, followed by incubation at 37 °C for 1 h. After incubation, the protein solution was removed, and the wells were washed twice with PBST. Then, AP-conjugated goat anti-mouse IgG was supplemented and incubated at 37 °C for 1 h. The unbound secondary antibody was discarded, and the wells were rinsed twice with PBST. Subsequently, 100 μL of PNPP chromogenic substrate (Seitz Biotech, Shanghai, China) was dispensed into each well, followed by incubation in the dark at ambient temperature for 30 min. The enzymatic reaction was halted by the addition of 50 μL of 3 mol/L NaOH to each well. Optical density was quantified at 450 nm utilizing a microplate spectrophotometer (Thermo Fisher Scientific). Negative controls consisted of serially diluted BSA standards (0, 3, 6, 9, 12, 15 μg), while a Tris-buffered saline formulation (50 mmol/L NaCl, 50 mmol/L Tris-HCl, pH 7.5) functioned as the blank.

### 2.5. Immunocytochemical Analysis

To visualize the cellular localization of AjTLR2 within coelomocytes following *V. splendidus* challenge, immunocytochemical staining was conducted according to established protocols [16,17]. Coelomic fluid was collected from dissected sea cucumbers and an equal volume of isotonic anticoagulant solution (20 mM EGTA, 68 mM Tris-HCl, 250 mM NaCl, and 19 mM KCl, pH 7.6) was combined with the sample. The mixture was filtered through a 200-mesh nylon gauze to remove large tissue debris, followed by centrifugation at 800 g for 10 min to harvest coelomocytes. An appropriate amount of isotonic buffer (1 mM EGTA, 10 mM Tris-HCl, and 443 mM NaCl, with pH stabilized at 7.6) was added to resuspend the cells, and the washing operation was repeated twice. After washing, the cell pellets were resuspended in L-15 working medium supplemented with 10 μL gentamicin sulfate, 50 μL penicillin-streptomycin mixture and 0.15 g NaCl. The cell suspension was seeded onto sterile coverslips and cell culture chamber slides, and incubated overnight at 16 °C. After removing the culture medium, the cells were subjected to three gentle washes with PBS prior to fixation with 4% paraformaldehyde for 20 min at ambient temperature. Upon fixation, the samples were immediately rinsed three times with PBS for 5 min each to completely eliminate residual fixative. Subsequently, cells were permeabilized with 0.5% Triton X-100 for 20 min under room conditions, and rinsed three times with PBS for 5 min each. After permeabilization, the wells were blocked with 5% bovine serum albumin (BSA) in TBST (20 mM Tris-HCl, 150 mM NaCl, 0.1% Tween-20, pH 7.5) for 60 min at room temperature. The blocking solution was aspirated directly without further washing. The primary antibodies, including mouse anti-AjTLR2 and rabbit anti-AjSRC antibodies, were diluted 1:200 in TBST, added to cell samples, and incubated overnight at 4 °C in a humidified chamber. On the next day, samples were washed three times with TBST for 5 min each. The corresponding fluorescent secondary antibodies, including Alexa Fluor 647-conjugated goat anti-mouse IgG (H+L) (Beyotime Biotechnology; Cat. No. A0473) and Cy3-conjugated goat anti-rabbit IgG (H+L) (Beyotime Biotechnology; Cat. No. A0516), were diluted 1:1000 according to the manufacturer’s instructions and incubated with the samples for 1 h at room temperature in the dark. After incubation, samples were rinsed three times with TBST for 5 min each. Following the final wash, DAPI staining solution was added to achieve a final concentration of 1 μg/mL, followed by 10 min of staining at room temperature and another three TBST washes. Finally, an appropriate volume of anti-fade mounting medium was dropped onto the slides, and coverslips were carefully mounted to avoid air bubbles. All sealed samples were maintained at 4 °C in the dark and observed and photographed promptly using a fluorescence microscope or laser scanning confocal microscope.

### 2.6. Determination and Quantification of ETs

ET formation was assessed in *A. japonicus* coelomocytes following stimulation with the indicated treatments. ET visualization was performed as previously described [18]. Briefly, cells were incubated with 5 μM SYTOX Green nucleic acid stain (Cat. NO. S7020, Thermo Fisher Scientific, Waltham, MA, USA) for 15 min, and nuclei were counterstained with DAPI. Fluorescence images were captured using a fluorescence microscope (LSM880, Zeiss, Oberkochen, Germany). For quantitative analysis, SYTOX Green fluorescence was measured with a fluorescence microplate reader (Tecan, Shanghai, China) at excitation and emission wavelengths of 485 and 525 nm, respectively.

### 2.7. RNA Interference Assays

GenePharma (Shanghai, China) was commissioned to design and synthesize lyophilized siRNAs specifically targeting AjTLR2 and AjSRC. A scrambled siRNA, exhibiting no sequence homology to any known gene in the *A. japonicus* transcriptome, served as the negative control (siNC). Given the instability of RNA, all manipulations were performed in a laminar flow hood using RNase-free pipette tips and centrifuge tubes. The lyophilized siRNA powder was centrifuged at 3500 rpm for 15 min, dissolved in 125 μL RNase-free water with vigorous vortexing to prepare a 20 μM working solution, and kept on ice until use. For transfection complex preparation, 10 μL Lipo6000 transfection reagent (Cat. NO. C0526, Beyotime, Shanghai, China), 10 μL siRNA, and 80 μL PBS were mixed in a centrifuge tube and incubated at room temperature for 15 min to form the transfection working solution. Individuals of *A. japonicus* received an injection of 100 μL of the aforementioned transfection reagent. Control individuals were injected with NC siRNA. Coelomocytes were collected at 24 h post-treatment for qPCR validation, and Western blot analysis was performed at 48 h post-treatment to evaluate the siRNA efficiency. Three biological replicates were set up for both the siRNA groups and the negative control group, respectively.

### 2.8. Pull-Down Assay

A pull-down assay was performed to identify proteins that interacted with the recombinant intracellular domain of AjTLR2 (rAjTLR2-IN). The coding sequence of the AjTLR2-IN domain was cloned into the pGEX-4T-1 vector (GE Healthcare, Chicago, IL, USA) and introduced into *E. coli* Rosetta competent cells. Subsequent affinity purification of the recombinant fusion protein was achieved using GST-Sepharose resin (GenScript, Nanjing, China). Recombinant GST-AjTLR2-IN was immobilized on GST Sefinose resin and incubated with protein extracts from coelomocytes of *A. japonicus* overnight at 4 °C. An equal volume of lysis buffer containing 2% (*v*/*v*) Triton X-100 in PBS was used as the negative control. Following incubation, the matrix was subjected to ten consecutive washes with a GST binding/wash buffer (Sangon Biotech, Cat. No. C600326) volume equivalent to 10 times that of the resin bed, ensuring the removal of unbound protein contaminants. Finally, one resin volume of GST elution buffer (Sangon Biotech, Cat. No. C600325) was added to collect the binding proteins, and this procedure was repeated three times. The collected elution fractions were separated and identified by SDS-PAGE, and the experimental group samples were compared with the control group samples. Bands that appeared exclusively in the experimental lane but not in the control lane were excised for further analysis of AjTLR2-IN-interacting proteins by LC-MS/MS. The pET-28a+ expression system was employed to produce fusion proteins capable of binding to AjTLR2-IN in *E. coli*. Incubation of GST-AjTLR2-IN with His-tagged proteins (1:1 molar ratio) was carried out at 4 °C for 6 h. After incubation with 50 μL of GST-bound resin at 4 °C for 6 h, the resin was washed 5 times with PBS. Bound complexes were displaced from the resin by the addition of GST elution buffer, consisting of 10 mM reduced glutathione and 50 mM Tris-HCl (pH 8.0). Concurrently, reverse His pull-down assays were conducted by incubating purified His-tagged proteins with GST-AjTLR2-IN, followed by adsorption onto Ni-NTA resin at 4 °C for 45 min. The immobilized complexes were subsequently washed five times with PBS. Bound complexes were displaced from the Ni-NTA matrix by applying a formulated elution system consisting of 0.5 M NaCl, 1 M imidazole, and 20 mM Tris-HCl (pH 8.0). Finally, the protein–protein interactions were analyzed by SDS-PAGE.

### 2.9. Quantitative Real-Time PCR Analysis (qPCR)

qPCR analysis was conducted to quantify the relative transcript abundance of the genes of interest. Total RNA from tissues or cells was extracted using the TRIzol method [19]. After verifying RNA purity and integrity via NanoDrop spectrophotometry and agarose gel electrophoresis, first-strand cDNA synthesis was carried out using 1 μg of total RNA and the PrimeScript RT Master Mix reagent (Cat. No. B300545, Sangon Biotech, Shanghai, China). Subsequently, amplification was carried out on an Applied Biosystems 7500 real-time PCR system using the SYBR Green I dye method with diluted cDNA as the template. qPCR mixtures (20 μL total) contained: 10 μL 2× TB Green Premix, 0.4 μL each primer (10 μM), 0.4 μL ROX dye, 2 μL cDNA, and 6.8 μL ddH_2_O. Amplification: 95 °C for 30 s, then 40 cycles of (95 °C for 5 s, 60 °C for 34 s). Melt curve: 95 °C for 15 s, 60 °C for 1 min, 95 °C for 15 s. The relative expression levels were calculated using the 2^−ΔΔCT^ method with β-actin for normalization [20]. All samples were run in three technical replicates.

### 2.10. Western Blotting

Western blot assay consisted of five main procedures, including gel preparation, protein electrophoresis, membrane transfer, antibody incubation, and signal detection. For gel casting, a one-step gel preparation kit (Cat. No. PG212, Epizyme Biotechnology, Shanghai, China) was used. The gel concentration was determined based on the molecular weight of the target proteins, and the sample comb was inserted accordingly. After gel solidification, the gel was mounted into the electrophoresis tank. Electrophoresis buffer was added, and equal amounts of total protein samples (normalized by protein concentration) together with molecular weight markers were subsequently loaded onto the gel. The initial electrophoresis voltage was set at 100 V. Once the bromophenol blue indicator migrated into the resolving gel, the voltage was increased to 150 V until the indicator reached the bottom edge of the gel. Upon completion of electrophoresis, the gel, PVDF membrane (pre-activated and equilibrated with methanol), and filter papers were assembled in a sandwich structure and placed in a transfer tank. Transfer was performed at 400 mA in the presence of rapid transfer buffer (Cat. No. WB4600, NCM biotech, Shanghai, China). The transfer duration was set from 20 to 40 min depending on the molecular size of proteins. Following protein transfer, the PVDF membrane was incubated in 5% (*w*/*v*) skim milk prepared in TBST for 2 h at room temperature for blocking, and then incubated with targeted primary antibodies overnight at 4 °C. On the following day, the membrane was washed with TBST buffer three times (10 min each time), and subsequently incubated with HRP-conjugated secondary antibodies at room temperature for 1 h. The secondary antibodies included HRP-conjugated goat anti-rabbit IgG and HRP-conjugated goat anti-mouse IgG antibodies (Beyotime Biotechnology, Shanghai, China; catalog numbers D110058-0100 and D110087-0100, respectively). After incubation with the secondary antibodies, the membrane was washed four additional times with TBST buffer (10 min per wash). Finally, ECL chemiluminescence substrate was added onto the membrane. After 2 min of reaction, imaging and photographing were performed using a chemiluminescence imaging system. The gray values of acquired images were analyzed via ImageJ software (version 1.53), and the relative expression level of target proteins was calculated as the gray value ratio of target protein to internal reference protein.

### 2.11. Statistical Analysis

Independent two-tailed t-test was employed to determine the statistical significance between two independent groups of data. For comparisons among multiple groups, one-way ANOVA combined with Bonferroni post hoc test was performed. Paired *t*-test was applied to analyze paired datasets consisting of control and experimental groups. A *p*-value below 0.05 was defined as statistically significant difference.

## 3. Results

### 3.1. Cloning, Sequence Analysis and Phylogenetic Tree of AjTLR2

**Figure 1:**
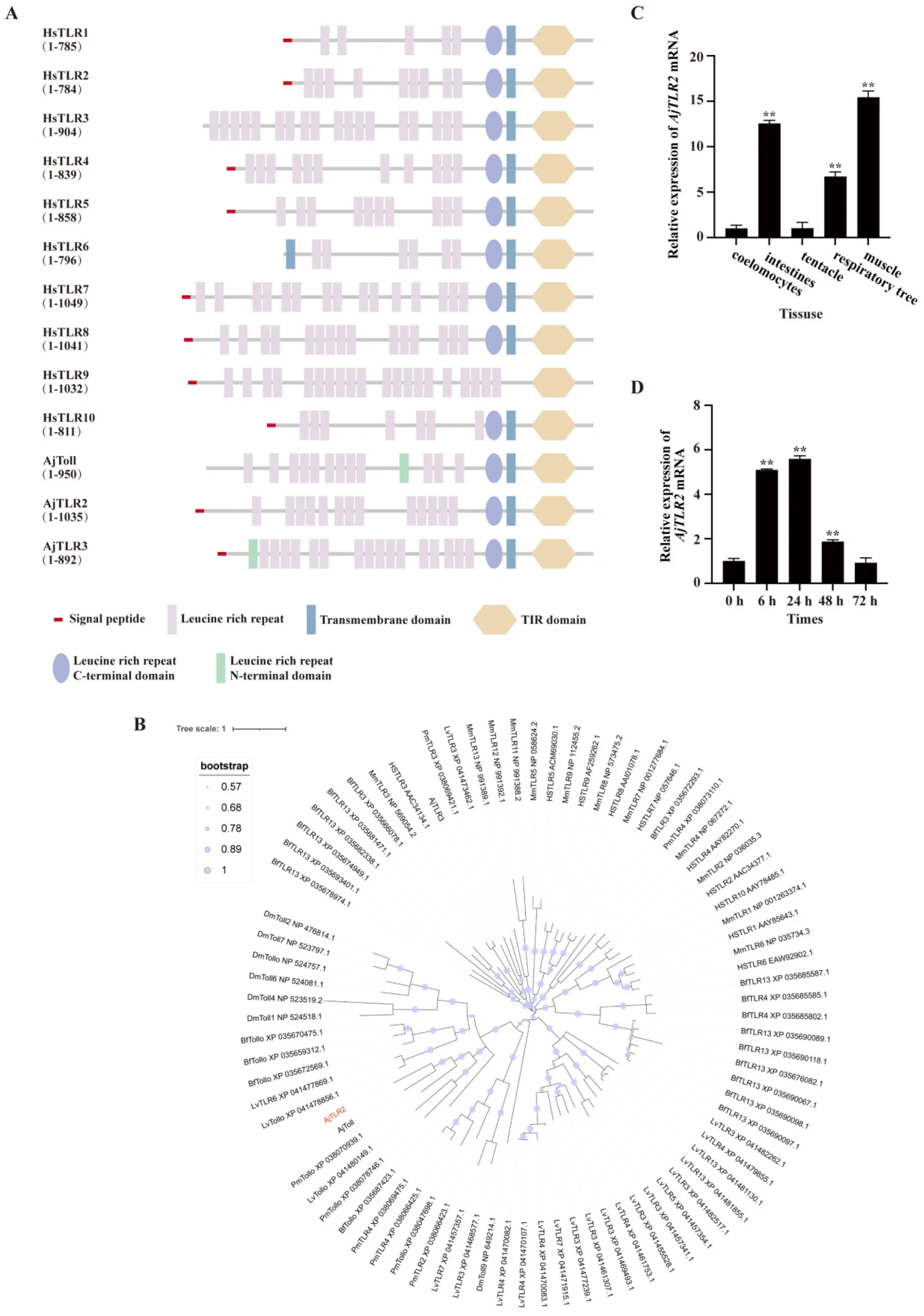
Molecular structural characteristics of AjTLR2. (**A**) SMART-based schematic comparison of domain topology between *A. japonicus* AjToll, AjTLR2, AjTLR3 and human HsTLR1–10. (**B**) Maximum-likelihood phylogenetic tree of Toll/TLR proteins from representative metazoans. Branch support values indicate bootstrap support from 1000 replicates. Species abbreviations are as follows: Aj, *Apostichopus japonicus*; Hs, *Homo sapiens*; Mm, *Mus musculus*; Pm, *Patiria miniata*; Lv, *Lytechinus variegatus*; Bf, *Branchiostoma floridae*; Dm, *Drosophila melanogaster*. Protein accession numbers are shown on the tree. (**C**) qPCR analysis of *AjTLR2* tissue expression patterns in healthy *A. japonicus*. Expression levels were normalized to the internal reference gene *Aj*β-actin and are presented relative to the expression level in coelomocytes. All values are shown as mean ± SD, with *n* = 5; asterisks indicate ** *p* < 0.01 for significant differences. (**D**) qPCR detection of *AjTLR2* mRNA expression in *A. japonicus* coelomocytes challenged with AJ01. All values are shown as mean ± SD, with *n* = 5; asterisks indicate ** *p* < 0.01 for significant differences.

A novel TLR (defined as AjTLR2) was identified in the sea cucumber *A. japonicus*. To characterize the newly identified AjTLR2 and provide a basis for subsequent functional analyses, we first analyzed its molecular features, evolutionary relationship, and expression profile in *A. japonicus*. Sequence analysis showed that the open reading frame of this gene contained 3108 base pairs and encoded 1035 amino acid residues (XP_071836949.1) (Figure S1). The typical Toll-family domain arrangement was identified in AjTLR2 via functional domain analysis, which consisted of an N-terminal signal peptide, an extracellular domain, a transmembrane region, and a C-terminal intracellular TIR domain. AjTLR2 contained 13 extracellular leucine-rich repeats (LRRs), and AjToll [21] contained 13, while AjTLR3 [21] contained 17 (Figure 1A). Moreover, the sequences of AjToll, AjTLR2 and AjTLR3 exhibited low sequence conservation. Discrepancies in both the count of extracellular LRRs and the sequence homology of cytoplasmic TIR domains imply that these TLRs exhibit responsiveness to diverse extracellular stimuli, including ligands and pathogens, thereby activating distinct intracellular signaling cascades. We performed phylogenetic analyses using four different tree-construction approaches: Maximum Likelihood (ML), Neighbor-Joining (NJ), Minimum Evolution (ME), and UPGMA. The resulting phylogenies showed generally consistent topological relationships, supporting the clustering of AjTLR2 with Toll/TLRs from other deuterostomes, including echinoderms and amphioxus, forming a distinct evolutionary lineage (Figure 1B and Figure S2A–C). In order to explore the potential biological functions of *AjTLR2*, we employed qPCR to survey the expression distribution of *AjTLR2*, normalizing data to the internal reference gene *Ajβ-actin*. The results presented in Figure 1C demonstrated a ubiquitous expression pattern of *AjTLR2* in the tested tissues. Quantitative analysis revealed that muscle tissue exhibited the peak expression level (15.44-fold, ** *p* < 0.01), followed sequentially by the intestine (12.56-fold, ** *p* < 0.01) and respiratory trees (6.70-fold, ** *p* < 0.01). The expression levels were calculated using the 2^−ΔΔCt^ method and normalized to the internal reference gene *Ajβ-actin*. To determine whether AjTLR2 responded to bacterial infection, the expression profile of *AjTLR2* in *A. japonicus* coelomocytes following AJ01 stimulation was investigated by qRT-PCR. The transcriptional level of *AjTLR2* was markedly elevated upon AJ01 stimulation, with the peak expression observed at 24 h post-infection (Figure 1D). Collectively, these results demonstrated that AjTLR2 was a conserved TLR that was broadly expressed in *A. japonicus* tissues and transcriptionally induced in coelomocytes following AJ01 challenge.

### 3.2. As a Membrane Receptor, AjTLR2 Recognizes Microbial Surface Components Through Its Extracellular LRR Domain

**Figure 2:**
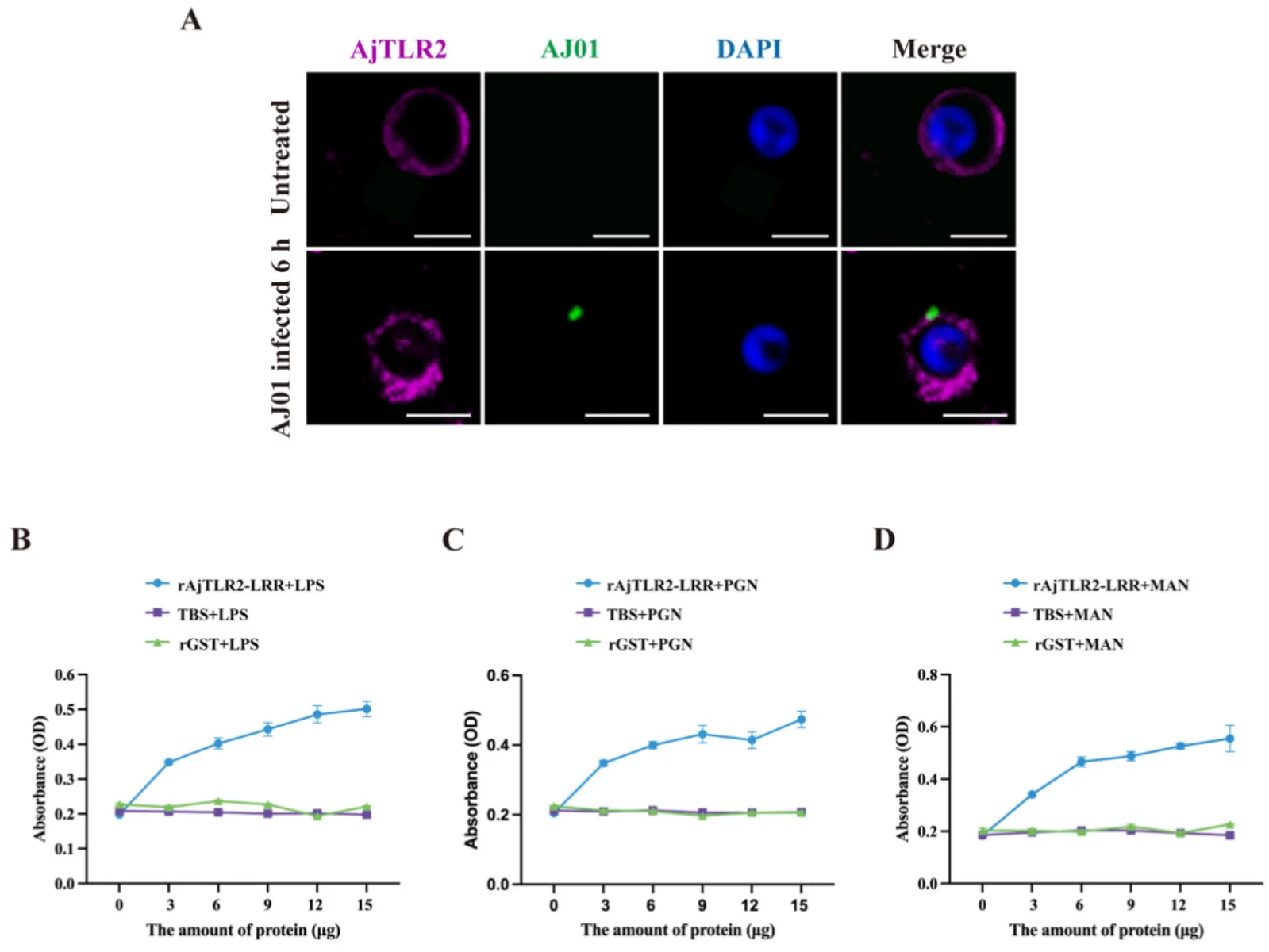
The LRR domain of AjTLR2 is responsible for immune recognition. (**A**) Fluorescence microscopy analysis of AjTLR2 (purple) in coelomocytes. AJ01 was stained with FITC (green). Nucleus was stained with DAPI (blue). Scale bar = 5 μm. (**B**–**D**) The binding capacity of rAjTLR2-EX toward different polysaccharide molecules was determined via ELISA. A total of four polysaccharides including LPS (*E. coli*), PGN and MAN were tested, with *n* = 5 for each treatment.

To determine whether AjTLR2 functions as a membrane-associated pattern recognition receptor, its subcellular localization and ligand-binding activity were examined. Immunofluorescence results of coelomocytes revealed that AjTLR2 localized to the cell membrane and colocalized with FITC-labeled AJ01 (Figure 2A). To determine whether cell surface polysaccharides were recognized by the extracellular region of AjTLR2, the binding of recombinant AjTLR2 extracellular domain (rAjTLR2-EX) to LPS, PGN, and mannan was evaluated by ELISA. The assays revealed that rAjTLR2-EX exhibited binding activity toward all tested ligands (Figure 2B–D). Collectively, these results demonstrated that AjTLR2 was a membrane-localized receptor capable of recognizing multiple microbial surface components through its extracellular domain.

### 3.3. AjTLR2 Regulates ET Formation in Coelomocytes After Recognizing AJ01

**Figure 3:**
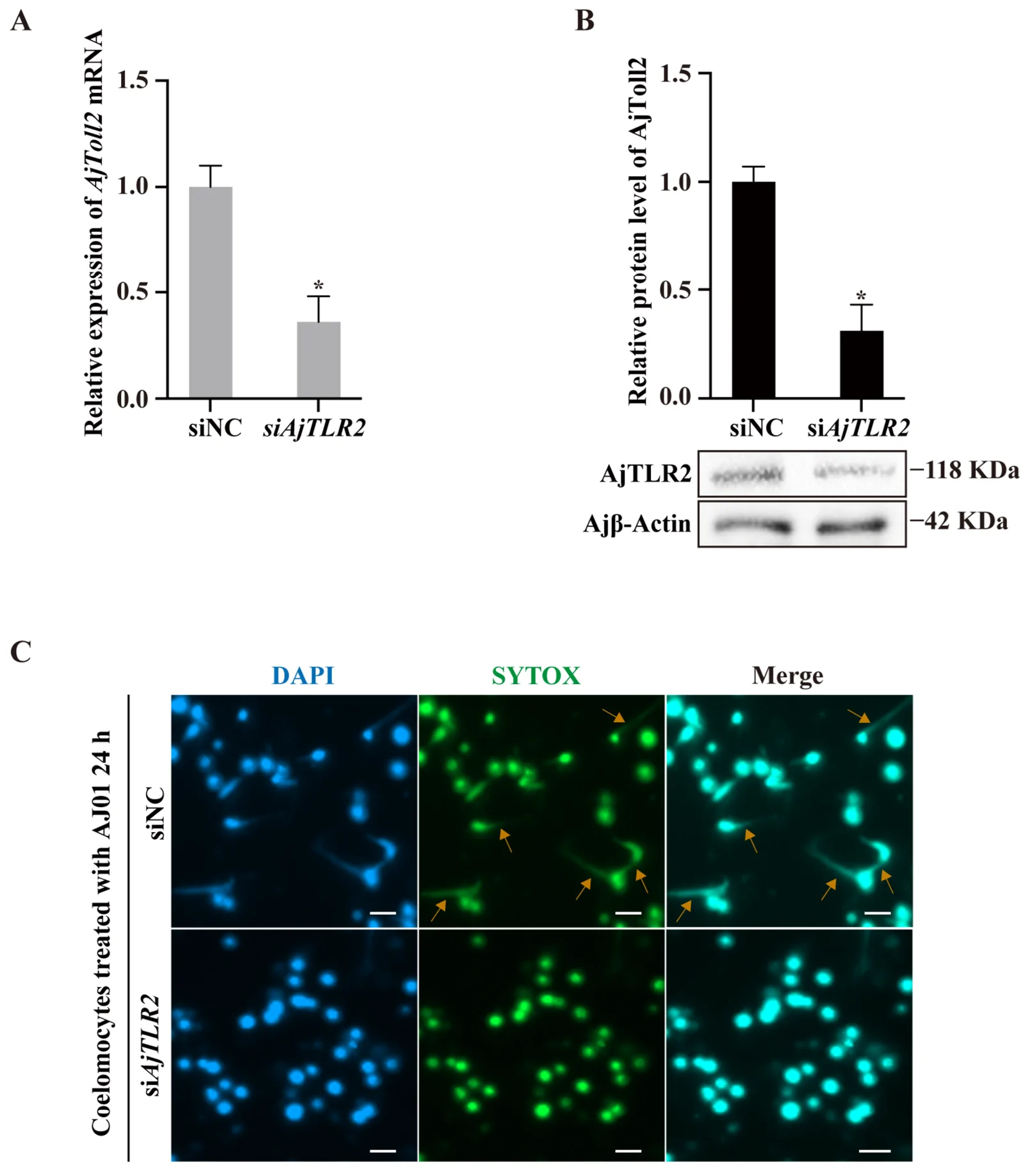
AjTLR2 mediates vital ETs. siNC indicates coelomocytes treated with non-targeting control siRNA, and si*AjTLR2* indicates coelomocytes treated with AjTLR2-specific siRNA. (**A**,**B**) The efficiency of si*AjTLR2* in coelomocytes was determined using qPCR and Western blot analysis. Values were given as mean ± SD, *n* = 5. Asterisks indicate significant differences: * *p* < 0.05. (**C**) ET formation was monitored via fluorescence microscopy in coelomocytes after siRNA treatment and AJ01 stimulation. SYTOX Green was used to visualize ET structures, and nuclear DNA was counterstained with DAPI (blue). Arrows denote the characteristic ET structures, scale bar = 20 μm.

AJ01 has been reported to induce the production of ETs in *A. japonicus* [12], but the regulatory mechanism remains unclear. In mammals, TLR2 has been implicated in the induction of ET formation [22]. Consequently, we explored whether AjTLR2 was involved in ET formation in *Apostichopus japonicus*. To this end, AjTLR2-specific siRNA (si*AjTLR2*) and non-targeting control siRNA (siNC) (Table 1) were utilized to achieve RNAi-mediated silencing of AjTLR2. Successful knockdown was confirmed by the marked reduction in both mRNA and protein levels after si*AjTLR2* transfection compared with the siNC group (Figure 3A,B). We next challenged the coelomocytes transfected with si*AjTLR2* or siNC with AJ01 for 24 h and observed ET formation in coelomocytes using confocal microscopy. The results showed that knockdown of AjTLR2 markedly reduced ET formation compared with the siNC group (Figure 3C). Collectively, these results demonstrated that AjTLR2 was required for AJ01-induced ET formation in *A. japonicus* coelomocytes.

### 3.4. AJ01 Flagellin C Is Specifically Recognized by AjTLR2, but Not by AjToll or AjTLR3

**Figure 4:**
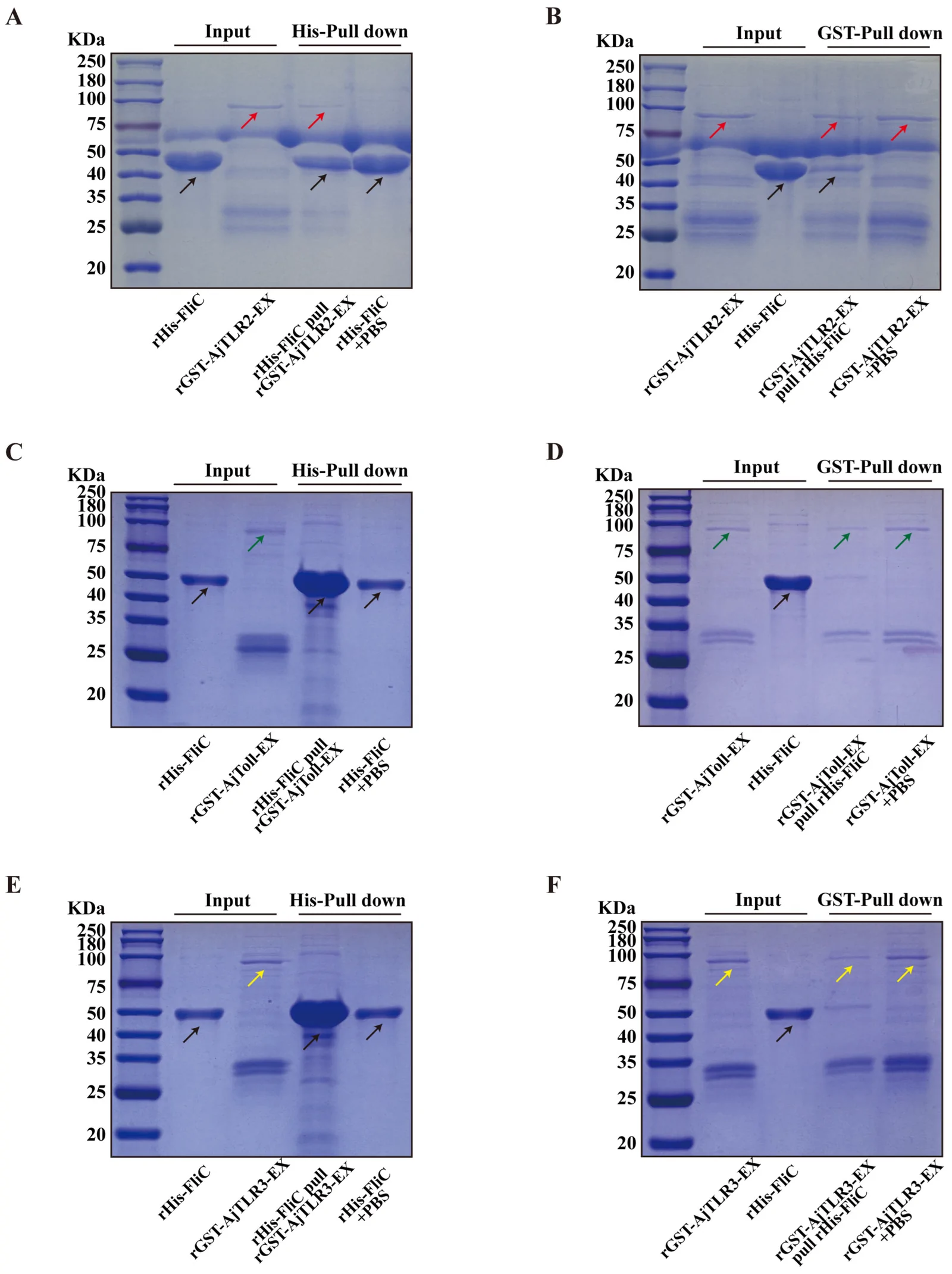
AjTLR2 specifically recognizes AJ01-FliC. (**A**,**B**) Pull-down assays were employed to detect the interactions between GST-tagged AjTLR2-EX and His-tagged FliC. The black arrow indicates the FliC protein band, while the red arrow indicates the AjTLR2-EX protein band. (**C**,**D**) Pull-down assays were employed to detect the interactions between GST-tagged AjToll-EX and His-tagged FliC. The black arrow indicates the FliC protein band, while the green arrow indicates the AjToll-EX protein band. (**E**,**F**) Pull-down assays were employed to detect the interactions between GST-tagged AjTLR3-EX and His-tagged FliC. The black arrow indicates the FliC protein band, while the yellow arrow indicates the AjTLR3-EX protein band. The pulled-down proteins were separated by SDS-PAGE under denaturing conditions and visualized by Coomassie Brilliant Blue staining.

The flagellin C of AJ01 has previously been shown to induce ET formation in *A. japonicus* coelomocytes [12]. To determine whether AJ01-FliC could serve as a ligand for AjTLR2, reciprocal GST and His pull-down assays were conducted to validate their direct interaction. As shown in Figure 4A, GST-tagged AjTLR2-EX (the extracellular LRR domain of AjTLR2) was specifically pulled down by His-tagged AJ01-FliC but not in the PBS control (rHis-FliC + PBS). Consistently, in the reciprocal GST pull-down assay (Figure 4B), His-tagged AJ01-FliC was efficiently pulled down by GST-tagged AjTLR2-EX but not in the PBS control (rGST-AjTLR2-EX + PBS), confirming the direct interaction between AJ01-FliC and the extracellular domain of AjTLR2. In vertebrates, flagellin is a specific ligand for TLR5 [23]. To further clarify the specific recognition of AjTLR2 for AJ01-FliC, we expressed recombinant LRR domains of two other TLRs from sea cucumber (AjToll and AjTLR3) and performed reverse GST and His pull-down assay with the recombinant protein of AJ01-FliC. The results showed that GST-tagged AjToll-EX did not interact with His-tagged AJ01-FliC (Figure 4C,D), nor did GST-tagged AjTLR3-EX interact with His-tagged AJ01-FliC (Figure 4E,F). Collectively, these results demonstrated that AJ01-FliC specifically interacted with the extracellular domain of AjTLR2 but not with those of AjToll or AjTLR3.

### 3.5. AjTLR2 Specifically Recognizes AJ01-FliC, Which Regulates the Formation of ETs

**Figure 5:**
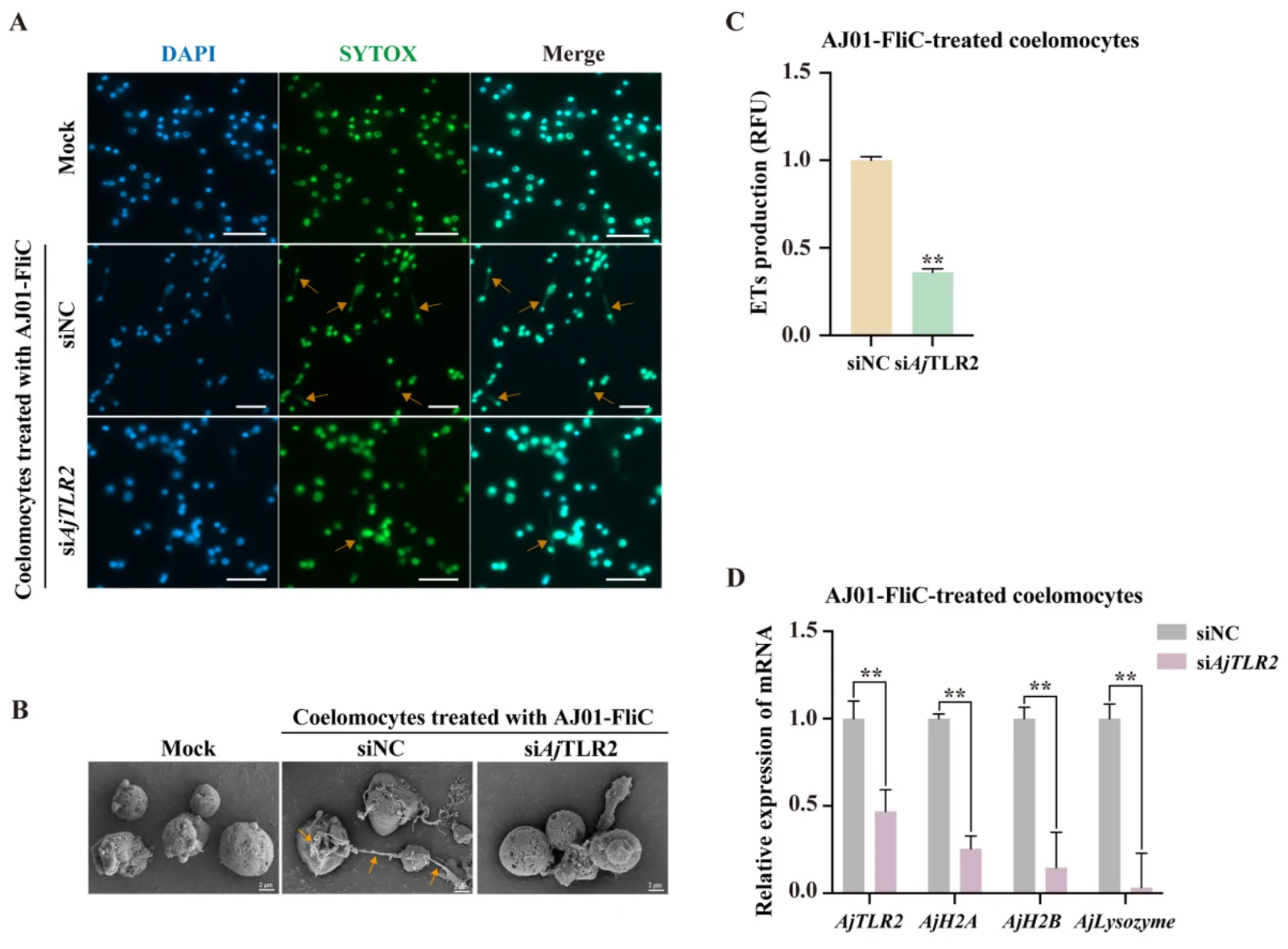
AjTLR2 regulates vital ETs by specifically recognizing AJ01-FliC of *Vibrio splendidus.* siNC indicates coelomocytes treated with non-targeting control siRNA, si*AjTLR2* indicates coelomocytes treated with AjTLR2-specific siRNA, mock represents cells treated without stimulation. (**A**) ET formation was monitored via fluorescence microscopy in coelomocytes after siRNA treatment and AJ01-FliC stimulation. SYTOX Green was used to visualize ET structures, and nuclear DNA was counterstained with DAPI (blue). Arrows denote the characteristic ET structures. Scale bar = 50 μm. (**B**) ET formation was observed by scanning electron microscopy (SEM) in coelomocytes after siRNA treatment and AJ01-FliC stimulation. Scale bar = 2 μm. (**C**) Quantification of the ETs. ET production was quantified as relative fluorescence units (RFU). (**D**) Expression analysis of *AjLysozyme*, *AjH2A* and *AjH2B* mRNA in coelomocytes after *AjTLR2* siRNA treatment and AJ01-FliC stimulation. Data are presented as the mean ± SD derived from five independent experiments. Statistical significance (** *p* < 0.01) is denoted by asterisks.

To clarify whether AjTLR2 regulates ET formation by recognizing AJ01-FliC. We examined the ET formation in coelomocytes after AJ01-FliC stimulation following AjTLR2 siRNA treatment. Immunofluorescence and SEM analyses revealed that the release of ET fiber structures was drastically diminished following AjTLR2 silencing compared with siNC group (Figure 5A,B). In order to achieve accurate detection of the amounts of ETs produced by stimulated coelomocytes, we quantified the ETs of coelomocytes using a fluorescence enzyme-linked immunosorbent assay (ELISA) reader (Figure 5C). AjTLR2-specific siRNA treatment reduced AJ01-FliC-induced ET production to 0.36-fold of that observed in the NC siRNA-treated group following AJ01-FliC stimulation (** *p* < 0.01). The quantitative analysis was consistent with the immunofluorescence and SEM observations. Histones are major structural components of ETs, together with antimicrobial proteins such as lysozyme [24]. We further examined the expression of the major components of ETs, including the histones AjH2A and AjH2B and the antimicrobial protein AjLysozyme, in coelomocytes using qRT-PCR. After knockdown of AjTLR2 expression, the transcript levels of *AjH2A*, *AjH2B*, and *AjLysozyme* were significantly decreased to 26%, 18%, and 3% of those in the siNC group, respectively (Figure 5D). Collectively, these results demonstrated that AjTLR2 mediated AJ01-FliC-induced ET formation in coelomocytes.

### 3.6. AjTLR2-Mediated ETs Require Interaction Between TLR Domains and SRC

**Figure 6:**
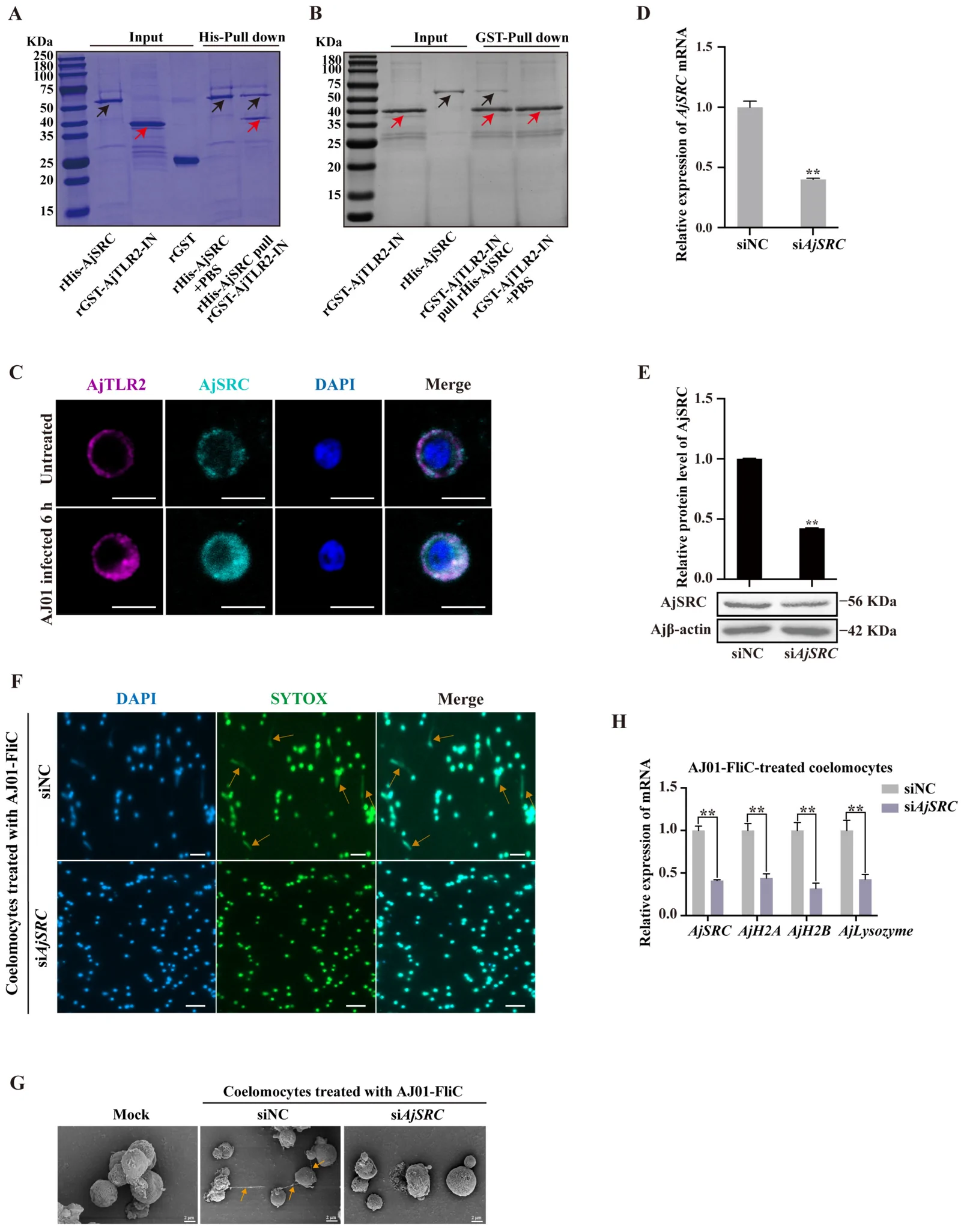
AjTLR2 mediates vital ET formation through the recruitment of AjSRC. (**A**,**B**) Pull-down assays were employed to detect the interactions between GST-tagged AjTLR2-IN and His-tagged AjSRC. The black arrow indicates the AjSRC protein band, while the red arrow indicates the AjTLR2-IN protein band. (**C**) Immunofluorescence staining was performed to explore the interaction of AjTLR2 and AjSRC, showing obvious co-localization between 647-AjTLR2 (purple) and Cy3-AjSRC (cyan), scale bar = 5 μm. (**D**,**E**) Both transcriptional (qPCR) and translational (Western blot) assays were used to confirm the silencing effect of si*AjSRC* on coelomocytes. Data are presented as the mean ± SD derived from five independent experiments. Statistical significance (** *p* < 0.01) is denoted by asterisks. (**F**) ET formation was monitored by fluorescence microscopy after siRNA treatment and AJ01-FliC stimulation, siNC indicates coelomocytes treated with non-targeting control siRNA, si*AjSRC* indicates coelomocytes treated with AjSRC-specific siRNA. SYTOX Green was used to visualize ET structures, and nuclear DNA was counterstained with DAPI (blue). Arrows denote the characteristic ET structures, scale bar = 50 μm. (**G**) SEM observation of ET formation after siRNA treatment and AJ01-FliC stimulation, mock represent coelomocytes treated without stimulation. Arrows indicate the ET structures, scale bar = 2 μm. (**H**) Expression analysis of *AjLysozyme*, *AjH2A* and *AjH2B* mRNA in coelomocytes after *AjSRC* siRNA treatment and AJ01-FliC immune stimulation. Data are presented as the mean ± SD derived from five independent experiments. Statistical significance (** *p* < 0.01) is denoted by asterisks.

Signal transduction after TLR activation depends on the recruitment of downstream adaptor proteins by its intracellular TIR domain [25,26]. To identify downstream signaling molecules involved in AjTLR2-mediated ET formation, proteins interacting with the intracellular TIR domain of AjTLR2 (AjTLR2-IN) were screened by pull-down assays coupled with MS/MS analysis. Through this screening, AjSRC was identified as a candidate interactor, a finding that was subsequently validated by reciprocal GST and His pull-down experiments (Figure 6A,B). Confocal microscopy revealed colocalization of AjTLR2 and AjSRC within coelomocytes, a phenomenon that was notably intensified following AJ01 challenge. (Figure 6C). These results demonstrated that AjTLR2 interacted with AjSRC and that their colocalization was enhanced following AJ01 stimulation. To explore the function of AjSRC during ET release, we first confirmed the silencing efficiency of AjSRC-specific siRNA (Figure 6D,E). We then observed ET formation in coelomocytes using immunofluorescence (Figure 6F) and SEM (Figure 6G). Similarly, we also measured the expression of ET-associated components (*AjH2A*, *AjH2B*, and *AjLysozyme*) after AjSRC siRNA treatment (Figure 6H). Collectively, these results demonstrated that AjSRC interacted with AjTLR2 and positively regulated AjTLR2-mediated ET formation in coelomocytes.

## 4. Discussion

ETs are important mechanisms by which cells prevent the spread of pathogens or handle larger volumes of microorganisms [27,28,29,30]. The mechanisms underlying ET formation have been extensively studied in higher organisms [22,24], but the activation and mechanism of ET formation in lower invertebrates still need to be elucidated. Here, we identify a novel Toll/TLR family receptor (AjTLR2) in sea cucumbers, which not only has broad PAMP and bacterial binding abilities, but also can recognize FliC specifically, promote the recruitment of AjSRC with its intracellular TIR domain, activate the downstream AjSRC signaling pathway, promote the expression of ET-associated components such as AjH2A and AjH2B, and mediate ET release to resist AJ01 infection.

The mechanisms underlying ET formation are complex, and different stimuli induce ET formation through distinct pathways. Based on current knowledge, ET formation occurs through multiple distinct mechanisms, including suicidal ETosis and vital ETosis, depending on the stimulus and signaling pathway [22,24]. Suicidal ETosis is characterized by plasma membrane rupture and cell death following chromatin decondensation, whereas vital ETosis allows extracellular trap release while maintaining plasma membrane integrity and cell viability [31,32]. Among them, an important characteristic of vital ETs is that their occurrence depends on cell surface receptors [11,33]. TLRs, as classic PRRs, play an important role in regulating NETs. Yipp et al. [9] report that non-suicidal NETs triggered by *Staphylococcus aureus* infection in mouse skin required the involvement of TLRs. Ting Wan et al. [10] also demonstrate that inhibitors of TLR2 and TLR4 significantly block *Staphylococcus aureus*-induced ET formation in vitro, whereas TLR2 and TLR4 agonists enhance ET formation. This is consistent with our findings that knocking down AjTLR2 expression significantly reduces ETs induced by *Vibrio splendidus*, indicating that AjTLR2 also plays a very important role in regulating ETs. In addition, we also find that AjTLR2 mediated release of ETs does not cause membrane rupture and cell death, which is consistent with the characteristics of vital ETs that distinguish them from the other two types of ETs [33]. The occurrence of ETs mediated by Toll/TLRs depends on their recognition of PAMPs and DAMPs. We find that AjTLR2 not only recognizes LPS, PGN, etc., like TLR2, but also specifically recognizes AJ01-FliC. As is well known, flagellin is a specific ligand for TLR5. A conserved sequence motif (XLXXXLXXNX + XXXX + XXX) has been identified within the LRR region of TLR5 and has been implicated in flagellin recognition [34]. However, we find this consensus sequence in all three Tolls/TLR of sea cucumbers, while in vitro pull-down experiments show that only AjTLR2 interacted with AJ01-FliC, whereas AjToll and AjTLR3 did not interact with AJ01-FliC. Therefore, the specific recognition of flagellar proteins by AjTLR2 may not only rely on classical consensus sequences, but its specific interaction mechanism deserves further exploration in the future.

When the receptor is activated, its extracellular domain recognizes PAMPs and DAMPs, triggering intracellular signaling cascades involving adaptor proteins, signaling complexes, and transcription factor complexes, ultimately leading to ET release. The conventional adaptor proteins for TLR are MyD88 or TRIF [6], but in this study, we find that AjSRC is recruited as a downstream signaling molecule for AjTLR2. Src family kinases (SFKs) can be activated by many cell membrane receptors, including TLRs, growth factor receptors, Fc receptors, and integrin signaling cascades [35]. A study by Ke Yang et al. [36] demonstrate in mouse macrophages that TLR4 acts as a core regulator of innate immunity, which recognizes LPS and further triggers downstream Src kinase signaling. When exposed to oxidized low-density lipoprotein on the cell membrane, TLR4 interacts with Src kinase to promote Src activation. Consistent with these findings, we find that recognition of AJ01-FliC by AjTLR2 promotes the recruitment of AjSRC, suggesting that Src-mediated signaling may also participate in the AjTLR2 signaling pathway. Furthermore, our pull-down assays demonstrate a direct interaction between AjSRC and the TIR domain of AjTLR2. Given that AjSRC contains a conserved SH2 domain, this interaction may be mediated by conserved structural domains; however, the precise molecular mechanism underlying the AjTLR2-AjSRC interaction requires further investigation. As a pivotal mediator of receptor-initiated signaling and cell–cell communication, Src functions as a master switch governing various downstream signaling routes. A series of studies show that Src plays a crucial role in regulating immune processes such as ET formation. Nanì et al. [37] suggest that PP2, an inhibitor of Src family kinases, effectively inhibits β-glucose-induced ET formation. Studies by Lu et al. [38] further demonstrate that genetic or pharmacological inhibition of Src markedly suppresses NET release, indicating an essential role for Src kinase in extracellular trap formation. In addition, Thompson-Souza et al. [39] indicate that the ET release induced by *Histoplasma capsulatum var. capsulatum* occurs through a mechanism dependent on the Src signaling pathway. Consistent with this finding, knockdown of AjSRC markedly inhibits AJ01-FliC-induced ET formation in *A. japonicus*, suggesting that the role of Src family kinases in extracellular trap formation may be evolutionarily conserved.

From an evolutionary perspective, our findings further expand the current understanding of TLR-mediated innate immunity in echinoderms. ETs have been reported in a variety of invertebrates, including crustaceans [40], mollusks [41], and annelids [42], indicating that ET formation represents an evolutionarily conserved innate immune defense mechanism. In vertebrates, ET formation can be initiated by a variety of PRRs, including TLRs, complement receptors, and both soluble and membrane-bound lectins [22]. In contrast, the involvement of PRRs in regulating ET formation remains largely unexplored in invertebrates [43]. Although TLR-mediated pathogen recognition has been extensively characterized in both vertebrates and invertebrates, direct evidence linking TLR signaling to ET formation is still limited.

## 5. Conclusions

In the present study, we demonstrate that AjTLR2 recognizes bacterial flagellin and recruits AjSRC to promote ET formation in *A. japonicus*. These findings provide new insights into the molecular mechanism underlying ET formation in echinoderms and further support the evolutionary conservation of TLR-mediated antimicrobial immunity across metazoans.

## Figures and Tables

**Table 1:** Sequences of siRNAs used in this study.

Primers	Sequence (5′-3′)	Used for
si*Aj*TLR2-1	CGAGCGUCAUAGAUUCAAAUU	RNA interference
si*Aj*TLR2-2	GGAAGCAGAUGUAAUUUAAAU	
si*Aj*SRC-1	GGUGAUGGCAAGCAUUUAATT	
si*Aj*SRC-2	GCGACCACGUCAAACAUUATT	
siRNA-NC (Negative control)	UUCUCCGAACGUGUCACGUTT	
	ACGUGACACGUUCGGAGAATT	

## Data Availability

Requests for access to the data, statistical code, questionnaires, and technical processes may be made by contacting the corresponding author at lichenghua@nbu.edu.cn.

## Supplementary Materials

The following supporting information can be downloaded at: https://www.mdpi.com/article/10.3390/biom16081097/s1, Figure S1: Sequence characters of AjTLR2; Figure S2: The tree was reconstructed using MEGA with Neighbor-Joining, Minimum Evolution, and UPGMA methods; Table S1: Sequences of primers used in this study.

## References

[1] Mushegian A., Medzhitov R. (2001). Evolutionary perspective on innate immune recognition. J. Cell Biol..

[2] Chen K., Zhang S., Shao Y., Guo M., Zhang W., Li C. (2021). A unique NLRC4 receptor from echinoderms mediates *Vibrio* phagocytosis via rearrangement of the cytoskeleton and polymerization of F-actin. PLoS Pathog..

[3] Kawai T., Ikegawa M., Ori D., Akira S. (2024). Review Decoding Toll-like receptors: Recent insights and perspectives in innate immunity. Immunity.

[4] Takeda K., Kaisho T., Akira S. (2003). Toll-like receptors. Annu. Rev. Immunol..

[5] Colleselli K., Stierschneider A., Wiesner C. (2023). An Update on Toll-like Receptor 2, Its Function and Dimerization in Pro- and Anti-Inflammatory Processes. Int. J. Mol. Sci..

[6] Bryant C.E. (2024). Rethinking Toll-like receptor signalling. Curr. Opin. Immunol..

[7] Alhamdan F., Bayarsaikhan G., Yuki K. (2024). Toll-like receptors and integrins crosstalk. Front. Immunol..

[8] Yamamoto M., Sato S., Hemmi H., Hoshino K., Kaisho T., Sanjo H., Takeuchi O., Sugiyama M., Okabe M., Takeda K. (2003). Role of adaptor TRIF in the MyD88-independent toll-like receptor signaling pathway. Science.

[9] Yipp B.G., Petri B., Salina D., Jenne C.N., Scott B.N.V., Zbytnuik L.D., Pittman K., Asaduzzaman M., Wu K.Y., Meijndert H.C. (2012). Infection-induced NETosis is a dynamic process involving neutrophil multitasking in vivo. Nat. Med..

[10] Wan T., Zhao Y.Y., Fan F.L., Hu R.J., Jin X.M. (2017). Dexamethasone Inhibits S-aureus-Induced Neutrophil Extracellular Pathogen-Killing Mechanism, Possibly through Toll-Like Receptor Regulation. Front. Immunol..

[11] Clark S.R., Ma A.C., Tavener S.A., McDonald B., Goodarzi Z., Kelly M.M., Patel K.D., Chakrabarti S., McAvoy E., Sinclair G.D. (2007). Platelet TLR4 activates neutrophil extracellular traps to ensnare bacteria in septic blood. Nat. Med..

[12] Shao Y., Lu T.Y., Shen S.K., Li C.H. (2025). MLKL promotes extracellular trap releasing in Vibrio splendidus AJ01-challenged coelomocytes of sea cucumber Apostichopus japonicus. Aquaculture.

[13] Lv Z.M., Li C.H., Zhang P.J., Wang Z.H., Zhang W.W., Jin C.H. (2015). miR-200 modulates coelomocytes antibacterial activities and LPS priming via targeting Tollip in Apostichopus japonicus. Fish Shellfish Immunol..

[14] Chen K.Y., Lv Z.M., Shao Y.N., Guo M., Li C.H. (2020). Cloning and functional analysis the first NLRC4-like gene from the sea cucumber Apostichopus japonicas. Dev. Comp. Immunol..

[15] Du X.J., Zhao X.F., Wang J.X. (2007). Molecular cloning and characterization of a lipopolysaccharide and β-1,3-glucan binding protein from fleshy prawn (*Fenneropenaeus chinensis*). Mol. Immunol..

[16] Sun J.J., Lan J.F., Zhao X.F., Vasta G.R., Wang J.X. (2017). Binding of a C-type lectin’s coiled-coil domain to the Domeless receptor directly activates the JAK/STAT pathway in the shrimp immune response to bacterial infection. PLoS Pathog..

[17] Chen K., Shen S., Lv Z., Guo M., Shao Y., Li C. (2025). Lytic coelomocyte death is tuned by cleavage but not phosphorylation of MLKL in echinoderms. PLoS Pathog..

[18] Poirier A.C., Schmitt P., Rosa R.D., Vanhove A.S., Kieffer-Jaquinod S., Rubio T.P., Charrière G.M., Destoumieux-Garzón D. (2014). Antimicrobial Histones and DNA Traps in Invertebrate Immunity EVIDENCES IN CRASSOSTREA GIGAS. J. Biol. Chem..

[19] Chomczynski P., Sacchi N. (2006). The single-step method of RNA isolation by acid guanidinium thiocyanate-phenol-chloroform extraction: Twenty-something years on. Nat. Protoc..

[20] Livak K.J., Schmittgen T.D. (2001). Analysis of relative gene expression data using real-time quantitative PCR and the 2^−ΔΔ*C*_T_^ method. Methods.

[21] Sun H., Zhou Z., Dong Y., Yang A., Jiang B., Gao S., Chen Z., Guan X., Wang B., Wang X. (2013). Identification and expression analysis of two Toll-like receptor genes from sea cucumber (*Apostichopus japonicus*). Fish Shellfish Immunol..

[22] Wang H., Kim S.J., Lei Y., Wang S.H., Wang H., Huang H., Zhang H.J., Tsung A. (2024). Neutrophil extracellular traps in homeostasis and disease. Signal Transduct. Target. Ther..

[23] Xia P.P., Wu Y.P., Lian S.Q., Yan L., Meng X., Duan Q.D., Zhu G.Q. (2021). Research progress on Toll-like receptor signal transduction and its roles in antimicrobial immune responses. Appl. Microbiol. Biotechnol..

[24] Wang Y.J., Du C.J., Zhang Y., Zhu L.L. (2024). Composition and Function of Neutrophil Extracellular Traps. Biomolecules.

[25] Sakaniwa K., Fujimura A., Shibata T., Shigematsu H., Ekimoto T., Yamamoto M., Ikeguchi M., Miyake K., Ohto U., Shimizu T. (2023). TLR3 forms a laterally aligned multimeric complex along double-stranded RNA for efficient signal transduction. Nat. Commun..

[26] Nimma S., Gu W.X., Maruta N., Li Y., Pan M.Q., Saikot F.K., Lim B.Y.J., McGuinness H.Y., Zaoti Z.F., Li S.L. (2021). Structural Evolution of TIR-Domain Signalosomes. Front. Immunol..

[27] Brinkmann V., Reichard U., Goosmann C., Fauler B., Uhlemann Y., Weiss D.S., Weinrauch Y., Zychlinsky A. (2004). Neutrophil extracellular traps kill bacteria. Science.

[28] Neumann A., Björck L., Frick I.M. (2020). Finegoldia magna, an Anaerobic Gram-Positive Bacterium of the Normal Human Microbiota, Induces Inflammation by Activating Neutrophils. Front. Microbiol..

[29] Kenny E.F., Herzig A., Krüger R., Muthu A., Mondal S., Thompson P.R., Brinkman V., von Bernuth H., Zychlinsky A. (2017). Diverse stimuli engage different neutrophil extracellular trap pathways. Elife.

[30] Ventura-Juarez J., Campos-Esparza M.R., Pacheco-Yepez J., López-Blanco J.A., Adabache-Ortíz A., Silva-Briano M., Campos-Rodríguez R. (2016). Entamoeba histolytica induces human neutrophils to form NETs. Parasite Immunol..

[31] Fuchs T.A., Abed U., Goosmann C., Hurwitz R., Schulze I., Wahn V., Weinrauch Y., Brinkmann V., Zychlinsky A. (2007). Novel cell death program leads to neutrophil extracellular traps. J. Cell Biol..

[32] Pilsczek F.H., Salina D., Poon K.K.H., Fahey C., Yipp B.G., Sibley C.D., Robbins S.M., Green F.H.Y., Surette M.G., Sugai M. (2010). A Novel Mechanism of Rapid Nuclear Neutrophil Extracellular Trap Formation in Response to Staphylococcus aureus. J. Immunol..

[33] Burgener S.S., Schroder K. (2020). Neutrophil Extracellular Traps in Host Defense. Cold Spring Harb. Perspect. Biol..

[34] Mizel S.B., West A.P., Hantgan R.R. (2003). Identification of a sequence in human toll-like receptor 5 required for the binding of Gram-negative flagellin. J. Biol. Chem..

[35] Lowell C.A. (2011). Src-family and Syk Kinases in Activating and Inhibitory Pathways in Innate Immune Cells: Signaling Cross Talk. Cold Spring Harb. Perspect. Biol..

[36] Yang K., Wang X.Q., Liu Z.H., Lu L., Mao J.Y., Meng H., Wang Y.N., Hu Y., Zeng Y., Zhang X.J. (2015). Oxidized Low-Density Lipoprotein Promotes Macrophage Lipid Accumulation via the Toll-Like Receptor 4-Src Pathway. Circ. J..

[37] Naní S., Fumagalli L., Sinha U., Kamen L., Scapini P., Berton G. (2015). Src Family Kinases and Syk Are Required for Neutrophil Extracellular Trap Formation in Response to β-Glucan Particles. J. Innate Immun..

[38] Lu G.T., Han F., Wang Y.D., Yuan C.C., Zhu Q.T., Xia T.Q., Chen L., Dong X.W., Ding Y.B., Xiao W.M. (2025). Src Reduces Neutrophil Extracellular Traps Generation and Resolves Acute Organ Damage. Adv. Sci..

[39] Thompson-Souza G.A., Santos G.M.P., Silva J.C., Muniz V.S., Braga Y.A.V., Figueiredo R.T., Melo R.C.N., Santos A.L.S., Pinto M.R., Neves J.S. (2020). Histoplasma capsulatum-induced extracellular DNA trap release in human neutrophils. Cell. Microbiol..

[40] Ng T.H., Chang S.H., Wu M.H., Wang H.C. (2013). Shrimp hemocytes release extracellular traps that kill bacteria. Dev. Comp. Immunol..

[41] Han Y.J., Chen L.Z., Zhang Q.Q., Yu D.D., Yang D.L., Zhao J.M. (2021). Hemocyte extracellular traps of Manila clam Ruditapes philippinarum: Production characteristics and antibacterial effects. Dev. Comp. Immunol..

[42] Homa J. (2018). Earthworm coelomocyte extracellular traps: Structural and functional similarities with neutrophil NETs. Cell Tissue Res..

[43] Cubillo-Martínez A.A., Pereyra M.A., Garfias Y., Guluarte C., Zenteno E., Sánchez-Salgado J.L. (2022). Extracellular traps involved in invertebrate immune mechanisms. Fish Shellfish Immunol..

