# Supplementary material for: Vibrio splendidus Flagellin C-Induced Extracellular Trap Release Relies on AjTLR2 Recognition in Apostichopus japonicus
Source: Biomolecules. 2026 Jul 27;16(8):1097. doi: 10.3390/biom16081097 (PMC13511744; doi:10.3390/biom16081097)

AjTLR2 sequence

```
        10         20         30         40         50         60
MKSILLHCHC NVTILFLLLN IISFGGCMVN ETICLQRWDC KWRTDFQYNH TEGQCSYLLP

        70         80         90        100        110        120
GYYPIDGPSS LAPEGSRCNL NVDCKRGDVN STEMFECTQL LTSQSLVESL RLYRCNNLEL

       130        140        150        160        170        180
KNGDLEFLTS MIEFNISYAV IKEMQDEVFY KTTNLVTVDV YQDYEAGVSI SEYPRAFIGP

       190        200        210        220        230        240
VPQSTSETSN TFLPNLRHLI LSSFNFSTGI PENAFQHLGA LQTLEFEFVT LQNSDFQSMQ

       250        260        270        280        290        300
NLTELAHLKI YDSQNIDSLP PRISTFMPNL QHFEISMTNI SNITVQQLES FHKLETLILD

       310        320        330        340        350        360
NNLLVNIDLL AFQHLTNLRS LSLAGNRQLQ IIPFFDRLTQ LEKLDLSNCA VSNLSLEVFS

       370        380        390        400        410        420
NLDNLRHLSL KKNKLRQIPP LFMNFPSITG TITLKNVEIV DLSDNEISSI MAYTFSNLEN

       430        440        450        460        470        480
LKSIDLYKNY ITVVHYRTFF NLKSLEKVSL EFNAIRNIHR EAFDGISTLK NLNLEGNMLK

       490        500        510        520        530        540
NFPKLSNVNW EVDLDHKLPR DPFSTDIRSN PLQCNCDMFR DVFTRVGRDI WQLQYYYDII

       550        560        570        580        590        600
WPGNYFLTNQ LFVQAKFLEC QTELYGKNKI DLMLHDPERW FSYVSSIDLC PHPCRCLSQC

       610        620        630        640        650        660
SNGHFYAVCR HANLTSIPNG LGSWVNTLYL EGNMITQLDS TSLSNMPDLT NVDLGDNRIS

       670        680        690        700        710        720
SIEDGTFHNL HSLREVSLRG NNLTVIHSST FNISSSQLKY LDISSNNIAD IDRDSFLSVS

       730        740        750        760        770        780
SLEVLKLDQN KLETISQGVF DNLHNLSLLT ISDNPFNCTC DLLYLTDWYV KVAFVPERSF

       790        800        810        820        830        840
SSDIAEIGCA PFLNETELYD WMQEQTVTCN PVIPPSDRPK PSSVIVVLVG VCAAAITSLV

       850        860        870        880        890        900
LGFIYKFRLE ILVLIYTKTG VKIFDRNDSN GEEEKTFDAF ISFSSMDRQF VLEEFIPRLE

       910        920        930        940        950        960
SEENKRKLCI HHRDFVVGEC IATNIINAIE NSNKILILCS KNYLESEWCS YEFKKSHHQA

       970        980        990       1000       1010       1020
LSDRSRRIVL IMMEDVNKST LDKEIKAYIS TNTYLEKKDP LFWSKLLYSV PAAKRGNIDT

      1030
LKETNANNVS SETCV
```

Figure S1. Sequence characters of AjTLR2. The signal peptide (1-27 aa), extracellular LRR domain (193-742 aa), transmembrane domain (823-845 aa), and TIR domain (876-1013 aa) are highlighted by red, green, orange, and blue boxes, respectively. The recombinant protein fragments used in this study are also indicated, including GST-AjTLR2-EX (137-791 aa; green dashed and solid boxes) and GST-AjTLR2-IN (870-1017 aa; blue dashed and solid boxes).

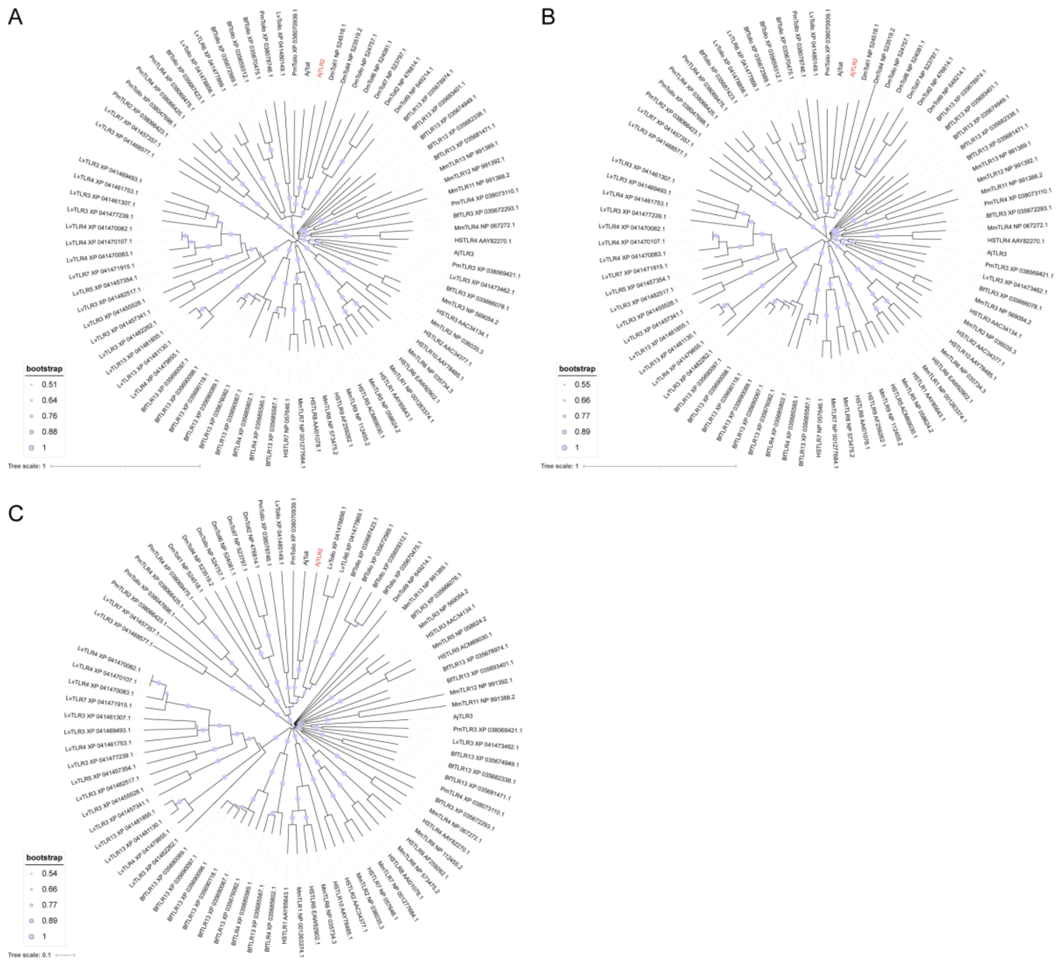

Figure S2. The tree was reconstructed using MEGA with Neighbor-Joining (A), Minimum Evolution (B), and UPGMA (C) methods. Branch support values indicate bootstrap support from 1,000 replicates. Internal nodes with bootstrap support values below 50% were collapsed into polytomies while retaining branch length information. Species abbreviations are as follows: Aj, *Apostichopus japonicus*; Hs, *Homo sapiens*; Mm, *Mus musculus*; Pm, *Patiria miniata*; Lv, *Lytechinus variegatus*; Bf, *Branchiostoma floridae*; Dm, *Drosophila melanogaster*. Protein accession numbers are shown on the tree.

Table S1. Sequences of primers used in this study

Primers	Sequence (5′-3′)	Used for
AjTLR2 F	ATGAAGTCGATTTTGCTTCATTGTC	Recombinant expression
AjTLR2 R	CTAAACACATGTTTCAGAAGAGACAT	
AjTLR2-EX F	TCATACGCTGTCATAAAGG	
AjTLR2-EX R	TGGAGCACATCCTATTTCTG	
AjTLR2-IN F	AACGGGGAAGAAGAGAAAACATTTG	
AjTLR2-IN R	GATATTGCCACGCTTAGCTGCTGGC	
AjSRC F	ATGGGTTGCCTAGGAAGCAAGAAGT	
AjSRC R	TTAGTTAGGCTCTGTGGACACAAAG	
AjToll-EX F	GGATCCGCGGGAAATTTACACAGTGGAGA	
AjToll-EX R	CTCGAGCAGTGGGTTTCCTCCTATGAAAACAG	
AjToll3-EX F	GGATCCAGCAGTCCTGCAGAAACTACTTCCTG	
AjToll3-EX R	CTCGAGTTTTCCATCACAGACATAGGGACTTG	
AjTLR2 qF	AACGGATTAGGCTCGTGGGTC	Real-time PCR
AjTLR2 qR	ATGAAAGGTGCCGTCCTCTAT	
AjSRC qF	TAGTAATGACATCCGCCCACC	
AjSRC qR	CTTCCTCCGTTCTTGCCTCAT	
Ajβ-actin qF	CCATTCAACCCTAAAGCCAACA	
Ajβ-actin qR	ACACACCGTCTCCTGAGTCCAT	
AjH2A qF	CGGTAACGCAGCAAGAGACAA	
AjH2A qR	GGGTAGAAGGACAGCCTGGATG	
AjH2B qF	ACAACAGGAAGTCCACCATCACC	
AjH2B qR	GGGGAGATAAGTCTGCCGTTTC	
AjLysozyme qF	ATGTCCCGTTACGCAACAGCCTC	
AjLysozyme qR	AGACCTTGCTCCAATAGCCATCG	

The original data of western blot

Fig. 3B IB：AjTLR2

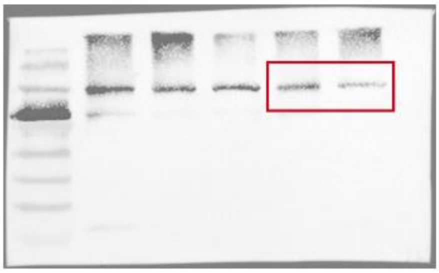

Fig. 3B IB：actin

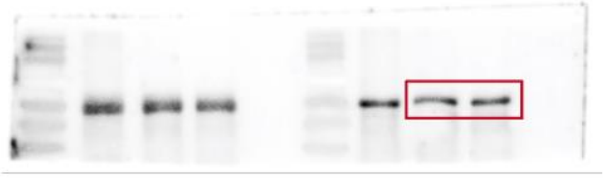

Fig. 6E IB：SRC

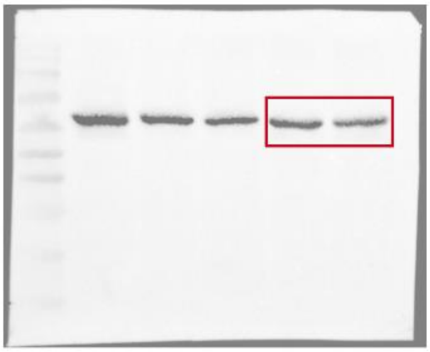

Fig. 6E IB：actin

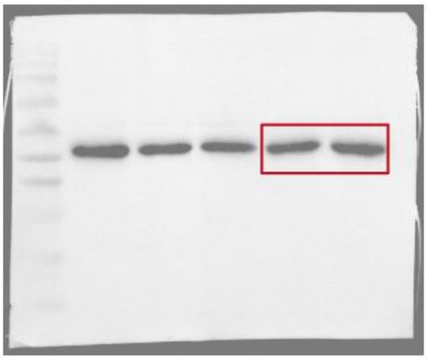

Supplement: Supplementary file 1 [file biomolecules-16-01097-s001.zip › biomolecules-4379581-supplementary.pdf]
